# Opinions and options about COVID-19: Personality correlates and sex differences in two European countries

**DOI:** 10.1371/journal.pone.0268193

**Published:** 2022-06-03

**Authors:** Sónia Brito-Costa, Peter Karl Jonason, Michele Tosi, Rui Antunes, Sofia Silva, Florêncio Castro

**Affiliations:** 1 Polytechnic Institute of Coimbra, Institute of Applied Research, Coimbra, Portugal; 2 Polytechnic Institute of Coimbra, Human Potential Development Center (CDPH), Research Group in Social and Human Sciences, Coimbra, Portugal; 3 Polytechnic Institute of Coimbra, Coimbra Education School, Coimbra, Portugal; 4 University of Padua, Padua, Italy; 5 University of Cardinal Stefan Wyszyński, Warszawa, Poland; 6 University of Estremadura, Badajoz, Spain; Universidad de la Republica, Facultad de Ciencias Sociales, URUGUAY

## Abstract

In the initial months of the COVID-19 pandemic in 2020, we collected data (N = 1,420) from Portugal and Spain in relation to personality (i.e., Dark Triad traits, Big Five traits, religiousness, and negative affect) and attitudes related to COVID-19 about its origins, opinions on how to deal with it, and fear of it. The most pervasive patterns we found were: (1) neurotic-type dispositions were associated with stronger opinions about the origins of the virus and leave people to have more fear of the virus but also more trust in tested establishments to provide help. (2): religious people were less trusting of science, thought prayer was answer, and attributed the existence of the virus to an act of God. We also found that sex differences and country differences in attitudes towards COVID-19 were mediate by sex/country differences in personality traits like emotional stability, religiousness, and negative affect. For instance, women reported more fear of COVID-19 than men did, and this was verified by women’s greater tendency to have negative affect and low emotional stability relative to men. Results point to the central role of neuroticism in accounting for variance in broad-spectrum attitudes towards COVID-19.

## 1 Introduction

In 2020 and 2021 most people’s social and professional lives were greatly disrupted with restrictions imposed by governments in attempts to minimize the spread and impact of COVID-19. Unsurprisingly, opinions were and continue to be extremely divided about the origins of the virus and what people should do in response. Researchers have already documented matters like who adheres to restrictions [1], specific behaviors like hoarding [2], psychosocial risk factors [3], the relationship between government policy compliance and psychological factors [4], and the psychological characteristics associated with hesitancy to take the COVID-19 vaccine [5]. In this study, we assess more general attitudes towards COVID-19 and relate them to a broad network of personality traits ranging from religiousness to psychopathy and test whether sex differences are a function of these traits. We do so in a sample of Portuguese and Spanish Facebook users, a medium that has proven essential for information and misinformation about the virus.

### Personality and COVID-19 attitudes

How people respond to and process information about an existential threat like COVID-19 should differ from person to person and (perhaps) the best way to understand how people differ from one another is to examine their personality traits. Personality traits may be behavioral expressions of latent emotional, cognitive, and perceptual biases [6]. They serve as generalized patterns of behavior that should be correlated with narrow-band features in the attitudinal landscape. Attitudes about COVID-19 are such narrowband attitudes; they are highly specific opinions, values, and motivations that relate to the threats posed by information about COVID-19 and nothing else. Here we build on previous research about COVID-19 by examining a wider array of narrow-band attitudes about COVID-19 in relation to personality traits.

First, we examine the Dark Triad traits of narcissism (e.g., grandiosity, entitlement), psychopathy (e.g., callous social attitudes, impulsivity), and Machiavellianism (e.g., pragmatism, deceitful). The traits had already studied extensively in relation to COVID-19. For instance, those high on these traits tend to engage in hoarding more, believe government measures will be ineffective at reduction [2], and to follow restrictions less [1]. However, these studies were highly focused in their assessment of attitudes towards COVID-19. Nevertheless, we expect some general trends. We expect those characterized by the Dark Triad traits:

1. be less fearful of it which may go some ways in explaining why they fail to follow restrictions, and,
2. to not think the best solution is to help others, to listen to the government, or prayer given their generally selfish [7], and “immoral” nature [8], along with their tendency to believe in conspiracies [9].

Second, we examine the role of religiousness. Religions are systems of beliefs that may have implications for attitudes and actions related to COVID [10, 11]. People who conform to the dicta of their faiths are, by definition, considered more religious [12]. For instance, those who are more religious (logically) should think that God created the virus but also think that prayer is the best answer to the infection. They may also distrust scientists and the medical establishment [13, 14] as ways to resolve the pandemic and given apparent overlaps (e.g., magical thinking) between conspiratorial thinking and religion [15], those who are religious may also believe the virus is a weapon created by the government, an ostensibly apocryphal belief. Moreover, some studies highlight the relationship between religiousness and the COVID-19 pandemic. People have spent more time praying during COVID-19 restrictions, people including those who have not been all that religious in the past [16]. Religious beliefs seem to enhance people’s sense of security when dealing with COVID-19 restrictions, leading to better emotional outcomes [17].

Third, we examine the role of emotional stability (from the Big Five traits) and dispositional negative affect as two central features of determinant of COVID-19 attitudes. People high on these traits are likely to be worrisome, anxious, depressed, and stressed [18, 19]. As dispositions, they may be undesirable and uncomfortable states, but in light of existential threats like COVID-19, they be useful alarm systems [20, 21]. As alarm systems, they should be sensitive to threats and enable belief systems and actions to minimize risk. As such, we expect an array of correlations with emotional stability and negative affect. For instance, people who lack emotional stability and have more negative affect should be:

1. more fearful of the virus which will serve to encourage them to take action like wearing masks or staying home [22, 23],
2. have generally stronger beliefs about the origins of the virus having spent more time ruminating about it,
3. to be unwilling to rely on luck or prayer as proven ineffective at treating medical conditions. Conversely, people with more emotional stability and less negative affect should (4) be more trusting in others, medicine, scientists, and governments as potential solutions because this may help them manage their feelings of existential dread.

### Sex differences, personality and COVID-19 attitudes

While personality traits are our primary focus here, we also explore the role of participant’s sex in accounting for individual differences in attitudes related to COVID-19. While controversial [24] and sometimes dismissed as sexist or trivial, participant’s sex may provide additional information about who holds certain attitudes about COVID-19 and allow us to test whether traits facilitate (i.e., mediate) sex differences in those attitudes. This will allow for a more nuanced, process-oriented approach where, instead of saying there are some sex differences and leave it at that, we explore the subsequent possibility that personality traits may serve as psychological mechanisms enabling men or women to hold particular attitudes.

First, we replicate sex differences in personality traits and examine whether there are sex differences in attitudes towards COVID-19. There is considerable evidence that men and women have different personality profiles [25, 26]. Two hypotheses attempt to account for these differences. Social role, learning, and constructivist accounts suggest that sex differences (or as these researchers might prefer, “gender” differences) in personality stem from sex-differentiated feedback from the world in behavior [27, 28]. These researchers contend that sex differences are trivial-to-small and are usually artifacts of some form of systematic oppression. For example, being selfish and aggressive—as seen in traits like narcissism—may be more strongly punished in girls than boys [29]. Alternatively, sex differences in personality might be expressions of recurrent, ancestral selection pressures for each sex and the traits serve as “solutions” to the asymmetries in costs and benefits of particular solutions. For example, being narcissistic might be more costly for women in the form of reproductive health problems [30] but more advantageous for men given increased sexual success [31].

Nevertheless, we expect:

1. men to score higher on the Dark Triad traits than women do [32], and:
2. women to be more extraverted, agreeable, conscientious, and neurotic (i.e., low emotional stability) than men are [33],
3. women to be more religious than men are [34],
4. and women to report more negative affective tendencies like depression, stress, and anxiety [35, 36].

Second, we explore the possibility that there are sex differences in attitudes towards COVID-19 [22]. As a rule, we expect women to have more dire or negative opinions about COVID-19. It serves as an existential threat, and women’s psychological systems may have been shaped more around avoiding risks than men’s [33]. This risk-aversion will have served women’s evolutionary needs more than men’s, and girls may be discouraged from taking risks over their development more than boys are. By having sensitive risk-aversion systems, women err on the side of caution and they, therefore, may have more aversive attitudes towards specific threats like COVID-19. However, to best test this, we need to also determine if sex differences in the attitudes towards COVID-19 are mediated by personality traits like neuroticism and negative affect which may serve as the psychological systems, that encourage women to avoid risk and personality traits like the Dark Triad traits that encourage men to engage in risk.

Here we report the results of a survey of Facebook users in Portugal and Spain about their opinions about COVID-19, the presence of sex/country differences in those attitudes, and the role of personality traits in understanding those effects. We quantify individual differences in how much fear they have, how much trust they have in the healthcare system or others, where they think the virus came from, and how they feel it is best to deal with it (e.g., prayer, listen to scientists).

## 2. Materials and methods

### 2.1. Participants and procedure

Participants (N = 1,420; 307 men, 1,113 women), 18 to 88 years (M = 43.40, SD = 14.81), invited to participate using Facebook advertising in Portugal (n = 1,034) and Spain (n = 386) to “take part in a personality study in relation to SARS-CoV-2” (i.e., COVID-19) in the early months of 2020 during the initial outbreak and when governments applied restrictions to mobility. The questionnaire was distributed from April to June 2020 to Portuguese and Spaniards who were members of an online group via Facebook. The questionnaire was parameterized to allow submission only with all questions answered, so there was no missing data that can generate other potential biases. The average time spent on responses was fifteen minutes for each participant. There were no payments to participants to encourage participation. On average, participants had a university degree (72%), were contract employees (59%), and were homeworking (45%). All subjects gave their informed consent, confirmed having read and understood and allow participate in the present study, were debriefed upon completion, and the protocol was carried out in accordance with the ethical and applicable regulations and guidelines of the Portuguese Psychologists Association as approved by the Ethics Committee of Infad. Data and supplemental files are located on the Open Science Framework: (https://osf.io/c9ean/?view_only=c3efaec049ff472da1ae6dfb35e92438).

### 2.2. Measures

To measure individual differences in the Dark Triad traits, we used the Spanish and Portuguese translations of the Dirty Dozen measure [7, 37, 38]. Participants were asked: how much they agreed (1 = not at all; 7 = very much) with 12 statements (4 per trait) such as: “I tend to want others to admire me” (i.e., narcissism), “I tend to lack remorse” (i.e., psychopathy), “I have used deceit or lied to get my way” (i.e., Machiavellianism), and items were averaged to create indices of each trait. The scale had fair-to-good internal consistency (see Table 2).

To measure individual differences in the Big Five traits we used the Spanish and Portuguese translations of the Ten Item Personality Inventory [39–41]. It is composed of ten items (2 per trait) where participant report how much (1 = not at all; 7 = very much) they think of themselves as “extraverted, enthusiastic” and “quiet, reserved” as measures of extraversion. The scale had fair internal consistency as measured with Spearman correlations given the ordinal nature of the data and the two items per trait (see Table 2).

To measure individual differences in the emotions of depression, stress, and anxiety we used the Spanish and Portuguese translations of the DASS-21 [19, 42–44]. It is composed of 21 items (7 per trait). Participants were asked: how much they agreed (0 = not at all; 3 = very much) with items like “couldn’t experience positive feeling” (i.e., depression), aware of dryness in mouth (i.e., anxiety), and “tended to over-react” (i.e., stress). Therefore, to minimize Type 1 error, we averaged the items to create one scale for negative affect (see Table 2) that had measurement invariance as a one-dimensional trait in the sexes and across countries (see online Appendix A).

To measure individual differences in religiousness with a single item in each language. As a narrow-band construct, a single-item measure should be sufficient. In English (but presented in Portuguese or Spanish depending on the sample. Participants were asked: how much they agreed (1 = not at all; 5 = very much) “do you consider yourself a religious person”.

To measure individual differences in how much people trusted the government’s health services: we created an ad hoc scale composed of three items, (i.e., “Do you trust the way in which the Government is managing the crisis situation imposed by the pandemic COVID-19?”; “Do you trust the response capacity of the National Health System?”; “Do you trust the responsiveness of Health Professionals?”), Participants were asked: how much they agreed (1 = not at all; 5 = very much). These items were averaged to create a single index of trust in healthcare services (α = .68).

To measure how much people thought the best solution was to just trust in other people: we used two ad hoc items (i.e., “Most citizens respect and comply with the protection measures imposed by the State of Emergency”; “Do you trust that most citizens will be able to act responsibly in this crisis situation?”), Participants were asked: how much they agreed (1 = not at all; 5 = very much). The two items were correlated (ρ = .74, p < .01) and averaged to measure what we will call trust in others.

To measure how much people believe COVID-19 would harm them, we created two ad hoc items (i.e., Do you think you will be harmed in your professional activity?”; “Are you afraid of being infected by COVID-19?”), where participants reported how much (1 = not at all; 5 = very much) each item applied to them. The two items were correlated (ρ = .13, p < .01) and averaged to measure what we will call fear of harm.

To measure people’s beliefs about the origins of COVID, we created three a hoc items (i.e., “The COVID-19 is a biological weapon manufactured in the laboratory”; “COVID-19 is a way for nature to rebalance itself”; and “COVID-19 is a message from God”) where participants reported how much (1 = not at all; 5 = very much) each item applied to them. We treated each as single-item indicators.

Then, to measure individual differences in how people deal with the pandemic, we created six ad hoc items (i.e., “the best way to deal with the pandemic is trust on luck”; “the best way to deal with the pandemic is study and know the problem”; “Do and act according to what the authorities/Government tell us”; “the best way to deal with the pandemic always see and hear information that the media make available to us”; “the best way to deal with the pandemic is pray and trust God”; “the best way to deal with the pandemic is being civically active”), where participants reported how much (1 = not at all; 5 = very much) each item applied to them. We treated each as single-item indicators.

## 3. Results

We began with a series of 2 (sex) × 2 (country) ANOVAs for the personality traits and our measures of COVID-related attitudes and opinions (see Table 1). Except for two potentially anomalous interactions for individual differences in religiousness (F[1, 1416] = 4.82, p <. 05, η2 < .01) and reliance on prayer as a solution (F[1, 1416] = 8.76, p < .05, η2 < .01), the differences in personality traits and attitudes related to COVID-19 were largely independently predicted by country and sex. Men scored higher than women in the Dark Triad traits, emotional stability, trust in others, and listening to scientists whereas women score higher than men in extraversion, conscientiousness, agreeableness, negative affect, religiousness, trust in others, thinking the virus was biological, natural, or supernatural in origin, listening to the government, and relying on prayer. Spaniards, compared to the Portuguese were more narcissistic, Machiavellian, emotionally stable, conscientious, trusting in healthcare and government, but were less extraverted, agreeable, open, negative in affect, religious, fearful of COVID-19, more likely to listen to the government and media, and to trust in prayer.

**Table 1:** Descriptive statistics, sex differences, and country differences for COVID-related beliefs, the Dark Triad traits, the Big Five traits, negative affect, and religiousness.

	Overall	Men	Women			Portugal	Spain		
*Personality*	*Mean* (SD)			*t*	*d*	*Mean* (SD)		*t*	*d*
Narcissism	9.92 (5.30)	11.17 (5.67)	9.57 (5.14)	4.75**	0.30	9.94 (5.14)	11.07 (5.55)	-5.06**	-0.30
Psychopathy	6.36 (3.43)	7.14 (4.06)	6.14 (3.20)	4.58**	0.29	6.34 (3.40)	6.40 (3.50)	-0.27	-0.02
Machiavellianism	6.08 (3.46)	6.99 (4.57)	5.83 (3.04)	5.21**	0.34	5.66 (3.01)	7.22 (4.24)	-7.68**	-0.46
Extraversion	9.14 (2.74)	8.36 (2.49)	9.35(2.76)	-5.70**	-0.37	9.39 (2.86)	8.47 (2.25)	5.67**	0.34
Agreeableness	10.77 (2.17)	10.50 (2.27)	10.85 (2.13)	-2.89**	-0.18	10.99 (2.11)	10.18 (2.22)	6.41**	0.38
Emotional Stability	8.79 (2.94)	9.90 (2.68)	8.48 (2.94)	7.60**	0.49	8.32 (2.84)	10.03 (2.87)	-10.09**	-0.60
Conscientiousness	10.80 (2.34)	10.50 (2.33)	10.83 (2.34)	-2.24*	-0.14	10.67 (2.31)	10.99 (2.41)	-2.30*	-0.14
Openness	10.40 (2.41)	10.32 (2.47)	10.40 (2.40)	-0.45	-0.03	10.48 (2.39)	10.11 (2.44)	2.58**	0.15
Negative Affect	14.61(13.68)	9.78(12.08)	15.94 (13.80)	-7.10**	-0.45	16.53 (13.88)	9.48 (11.67)	8.86**	0.52
Religiousness	1.93 (0.83)	1.74 (0.83)	1.98 (0.82)	-4.51**	-0.29	2.01 (0.82)	1.72 (0.82)	5.90**	0.35
***COVID measures***									
Trust in healthcare	10.29 (2.64)	10.49 (2.76)	10.24 (2.61)	1.44	0.09	10.10 (2.64)	10.82 (2.58)	-4.63**	-0.27
Trust in others	5.96 (1.89)	6.42 (1.83)	5.83 (1.87)	4.92**	0.31	5.65 (1.78)	6.77 (1.88)	-10.33**	-0.62
Fear of COVID	6.50 (1.93)	6.06 (1.93)	6.62 (1.91)	-4.48**	-0.29	6.77 (1.85)	5.77 (1.95)	8.90**	0.53
**Origins of COVID**									
-Biological Weapon	2.64 (1.36)	2.42 (1.35)	2.70 (1.36)	-3.27**	-0.21	2.65 (1.35)	2.61 (1.38)	0.58	0.03
-Naturally Occurring	2.73 (1.31)	2.44 (1.30)	2.81 (1.30)	-4.42**	-0.28	2.79 (1.33)	2.57 (1.24)	2.92**	0.17
-God Created It	1.66 (1.10)	1.47 (0.99)	1.71 (1.13)	-3.31**	-0.21	1.74 (1.15)	1.43 (0.92)	4.81**	0.29
**How to deal with it**									
-Rely on luck	1.66 (1.05)	1.66 (1.09)	1.66 (1.03)	0.07	0.00	1.66 (1.04)	1.67 (1.06)	-0.11	0.00
-Listen to scientists	4.54 (0.89)	4.65 (0.81)	4.51 (0.92)	2.39*	0.15	4.52 (0.87)	4.61 (0.97)	-1.76	-0.10
-Listen to the government	4.27 (0.95)	4.14 (0.94)	4.39 (0.94)	-2.67**	-0.17	4.37 (0.89)	3.98 (1.04)	7.07**	0.42
-Listen to the media	3.52 (1.12)	3.50 (1.06)	3.53 (1.13)	-0.31	-0.02	3.69 (1.07)	3.09 (1.13)	9.27**	0.55
-Prayer	2.41 (1.44)	2.08 (1.40)	2.51 (1.43)	-4.61**	-0.27	2.54 (1.43)	2.08 (1.41)	5.36**	0.32
-Help others	4.31 (0.96)	4.38 (0.88)	4.29 (0.99)	1.51	0.09	4.28 (0.95)	4.38 (1.00)	-1.68	-0.10

*Note*. Cohen’s *d* was calculated online (https://lbecker.uccs.edu/).

* *p* < .05

** *p* < .01

In Table 2 we report the correlation between the personality traits and COVID-19 scales/items using Spearman’s ρ which were nearly identical to Pearson’s rs in size and direction but allowed us to deal with normality violations which may be especially pronounced in brief and single-item measures [45, 46]. Those who felt people should trust in healthcare systems were more narcissistic and emotionally stable and less depressed, anxious, stressed, and religious. Those who felt people should simply trust in others to do the right thing were more narcissistic, open, emotionally stable, and conscientious and were less extraverted and with less negative affect.

**Table 2:** Correlations (ρ) between COVID-related beliefs and the Dark Triad traits, the Big Five traits, depression and anxiety scores, and religiousness along with estimates of internal consistency.

	Dark Triad			Big Five						
*COVID measures*	N	P	M	O	C	E	A	Es	Na	R
Trust in healthcare	.09**	-.02	.05	.01	.04	< .01	< .01	.14**	-.16**	-.15**
Trust in others	.10**	-.01	.05	.07**	.08**	-.05*	< .01	.23**	-.29**	-.09
Fear of COVID	-.07**	-.03	-.07**	-.03	-.05	.02	< .01	-.25**	.33**	.16**
**Origins of COVID**										
-Biological Weapon	-.10**	-.02	-.02	-.02	.07**	.03	.03	-.13**	.15**	.24**
-Naturally Occurring	-.03	< .01	.02	.02	-.01	.02	< .01	-.08**	.13**	.12**
-God Created It	-.06*	< .01	-.03	-.06*	-.08**	< .01	.05	-.10**	.22**	.43**
**How to deal with it**										
-Rely on luck	< .01	.13**	.06*	-.10**	-.07**	-.04	-.06*	-.12*	.15**	.13**
-Listen to scientists	.07**	-.12**	-.04	.08*	.07**	.03	< .01	.12**	-.14**	-.15**
-Listen to the government	-.01	-.11**	-.10**	.06*	.04	.10**	.16**	-.04	< .01	< .01
-Listen to the media	< .01	-.04	-.07**	.10**	.02	.05	.13**	-.03	< .01	.08**
-Prayer	-.09**	-.08**	-.10**	-.08**	-.01	< .01	.17**	-.09**	.24**	.70**
-Help others	-.05	-.15**	-.13**	.16**	.08**	.10**	.15**	.07**	-.11	-.01
Cronbach’s α	.85	.68	.83	.21	.25	.36	.21	.43	.95	--

Note.: N = narcissism, P = psychopathy, M = Machiavellianism, O = openness, C = conscientiousness, E = extraversion, A = agreeableness, Es = emotional stability, Na = negative affect, R = religiousness. Internal consistency was assessed with Spearman ρ for the Big Five scales because they were composed of only two items.

* p < .05

** p < .01

More fear of COVID-19 was associated with less narcissism, Machiavellianism, and emotionally stability but more negative affect and religiousness. Beliefs that COVID-19 was a biological weapon were associated with less narcissism and emotionally stability but more negative affect, and religiousness. Beliefs that COVID-19 was naturally occurring were associated with less emotionally stability but more negative affect and religiousness. Beliefs in the divine origins of COVID-19 were associated with less narcissism, openness, conscientiousness, and emotionally stability but more negative affect and religiousness.

Thinking that luck is the best option was associated with more psychopathy, Machiavellianism, negative affect, and religiousness but less openness, conscientiousness, agreeableness, and emotional stability. Thinking it is best to listen to scientists was associated with more narcissism, openness, emotional stability, and conscientiousness, but less psychopathy, negative affect, and religiousness.

Beliefs that listening to the government was best were associated with less psychopathy and Machiavellianism but more openness, extraversion, and agreeableness. Beliefs that it as best to listen to the media were associated with less Machiavellianism but more openness, agreeableness, and religiousness. Beliefs that prayer was the best solution were associated with less of all the Dark Triad traits, openness, and emotional stability, but more agreeableness, negative affect, and religiousness. Beliefs that it was best to simply help others were associated with less psychopathy, Machiavellianism, negative affect, but higher scores on all the Big Five traits.

Based on criteria set out by Baron and Kenny [47], we reduced the total number of mediation tests for sex differences in COVID attitudes as a function of the personality traits. We tested combined multiple mediation effects and the influence of each mediator one at a time (see S1 Table). Sex differences in trust in others, fear of COVID-19, and beliefs that COVID-19 was a biological weapon, created by God, or naturally occurring were mediated by emotional stability, negative affect, and religiousness, suggesting that being more disposed to these personality traits as women leads them to be more likely to hold these beliefs. Sex differences in beliefs that listening to scientists was the best option were mediated by psychopathy, Machiavellianism, and religiousness, suggesting being lower in psychopathy and Machiavellianism, and less religiousness, facilitated less trust in scientists. Sex differences in listening to the government were mediated by psychopathy, Machiavellianism, extraversion, and agreeableness, suggesting being lower in the former and higher in the latter, facilitated the belief in listening the government. And sex differences in believing prayer was the best option were mediated by narcissism, agreeableness, negative affect, and religiousness, suggesting being lower in narcissism, and higher in the other traits, facilitated this belief in women.

Lastly, we explored (no a priori hypotheses) potential mediation of country-level differences in the COVID attitudes (see S1 Table) as a function of the personality traits. Again, we tested combined multiple mediation effects and the influence of each mediator one at a time (see S2 Table). Spanish was more likely to trust in healthcare and showed higher emotional stability, lower in negative affect, and less religious than those from Portugal. Similarly, thinking people need to trust in others in response to COVID-19 were more common in Spaniards shown more emotional stability and less negative affect than Portuguese. Spanish people thought COVID-19 was naturally occurring and showed less emotional stability and lower in negative affect than Portuguese. Spanish showed lower tendency to think one should listen to the government and higher rates of Machiavellianism and less extraversion and agreeableness. Spanish were less likely to think one should listen to the media and were less agreeable than the Portuguese. And last, Spaniards were less likely to believing in prayer was the best option and were higher in narcissism, and lower in openness, agreeableness, negative affect, and religiousness.

## 4. Discussion

We write this passing the one-year mark of restrictions imposed by national governments in attempts to reduce the spread and severity of COVID-19. Over the last year, it has become evident that this is a multifaceted issue where considerable disagreements exist about where the virus came from, what to do about it, and how much fear people have about it. As such, our estimation of attitudes towards the virus were commensurately complex and varied unlike other studies that were more narrowband, examining, for instance, compliance with restrictions and hoarding [1, 2]. Given this, however, we found many correlations that were larger than expected by chance which we will refrain from discussing in hopes of centering our discussion on larger issues. Therefore, we focus here on the larger ones (> .10) to reduce Type 1 error inflation and to ease interpretation of the effects.

The central revelations here were that across a wide array of attitudes towards COVID-19 (1) women were more likely to hold stronger, wider, and more cautionary attitudes about the virus and (2) this was enabled by both neuroticism (i.e., negative affect and low emotional stability) and religiousness. Neurotic people may be hypervigilant to threats [4, 48]), experiencing more psychopathologies and psychosomatic complaints [49] because they have an overactive limbic system [50]. On the other hand, neuroticism is most associated with poor mental health, and extraversion is associated with a reluctance to socially isolate. Conscientiousness predicts compliance with safety guidelines but also with fewer prosocial behaviors, particularly stockpiling [51]. COVID-19 may be one such threat for woman because their ancestrally heightened perilous lives compared to men [52–55]. As dispositions, they may be undesirable and uncomfortable states, but in light of existential threats like COVID-19, they be useful alarm systems [20, 21]. Neuroticism may serve to expedite and focus people—mostly women’s—psychological systems to avoid threats, pursue sensible people and practices to reduce exposure, and to generally have spent more time considering the nature of the virus. Simultaneously, however, women were more likely to be religious [34] which may disable their tendencies to take appropriate and effective acts to minimize risk with beliefs that prayer might be the best option and distrust of scientists [13, 14]. Taken together, while women have different attitudes about COVID-19 than men do, the outcomes those women experience may be a function if they live a cautious life or a religious one. And yet, if the outcomes are focused on emotional management both neuroticism and faith may play a central role [17].

Beyond the central role of neuroticism and religiousness, there were smaller and less systematic effects for the Dark Triad traits and the remaining Big Five traits along with country-level effects. We generally replicated sex differences in the traits like men scored higher on the Dark Triad traits than women did [32] and replicated various sex differences with the Big Five traits [33]. We found that the people with risk-prone traits of the Dark Triad traits were associated with less fear and less reliance on prayer, luck, and others. These may reflect tendencies of those who are characterized by the Dark Triad traits to be antisocial and amoral but also to cynical or pessimistic [56]. Alternatively, open and conscientious people were more likely to trust others, less likely to think God created COVID-19, less likely to rely on luck, and more likely to trust scientists. These may reflect education/liberal effects in terms of socio-political orientation. Nevertheless, the effects for the Big Five and the Dark Triad traits were all rather weak so making too much of them should be avoided. Similarly, country differences were present in personality and COVID-19 attitudes and personality differences mediated some of these country-level effects however, we failed to capture ostensible mechanisms that might explain why Spain and Portuguese participants might vary. We cannot rule out sampling errors here, so strong interpretations should be avoided here as well. However, until now, there is no consistent relation between aversive personality and negative effect regarding the pandemic [57].

While our data comes from two understudied populations relative to Western samples, captures a wide array of traits, emotions, and COVID-related attitudes, our study is limited, since those results could be affected by the sociocultural context and sociodemographic factors, and by the differences in life circumstances that could plausibly affect risky decision making in an ongoing pandemic. Nonetheless, First, we relied heavily on ad hoc measures of COVID-attitudes and brief measures of personality. We did so to maximize breadth but, for some researchers, this may mean we have unreasonably sacrificed validity in our measurement. Future research may—if the virus continues or returns—replicate our findings with “superior” measures. Nevertheless, the measures are not terribly problematic given (as reported on the OSF site) its alignment with prior research about, for instance, the Dirty Dozen measure [32, 58] which has been validated in, for instance, Spanish [59]. Second, our sample in Portugal was four times larger than the sample in Spain which could mean that our results were more about the former than the latter but given the lack of moderation and the fact that the Spanish sample is similar in size to most single-country research on personality psychology, we are confident in our results. Third, given the large overall sample size, many of our effects were apparently small. Indeed, most of the effects could be considered small (i.e., r < .20), meaning that many of our effects might be either trivial or error. Assuming they are not error, even small effects can have major implications for populations at large (i.e., a single person is needed to spread viruses around) and, thus, our results have potentially important epidemiological implications.

Fourth, our use of the DASS measure was potentially problematic in that we failed to use its three dimensions and, instead, used a single dimension we called “negative affect”. While the three-dimensional model fit the data slightly better than the one-dimensional model, the correlations between the three aspects were problematically high (rs = .72-.78), the results aligned 100% of the time, and the internal consistencies within each (αs = .89-.92) and overall (α = .95) suggest the scale might need further refinement or be uniquely affected by the COVID situation. In terms of measurement, the scale appears to be bloated-specific—vis-a-vis the internal consistencies. Our failure to find different outcomes associated with each may mean the three aspects are making distinctions without differences. This logical fallacy violates Ockham’s Razor, which the use of a single dimension reduces (along with Type 1 error inflation). However, prior research has found utility in the three dimensions operating as independent traits [60, 61] so, perhaps, it is sampling error or is situational. Alternatively, the pandemic may threaten people enough that it aligns their negative affective states towards self-protection. Evolutionary psychologists think that the functional utility of negative affective states like worry, depression, and neuroticism may serve as alert systems [20, 21]. Such a contention could be tested by comparing DASS scores and its factor structure in threating and non-threating conditions (e.g., via priming) or across broader situational ratings. In other hand, having cross-sectional data limits the ability to draw causal conclusions. We encourage future studies under the proposed methodology to check if cultural differences and demographics domain have influence on the results. Also future longitudinal studies will allow the possibility to evaluate the adaptive or maladaptive nature of individuals, which would be very interesting and an advance for the literature.

## 5. Conclusions

Despite these limitations, we have provided new details about people’s attitude towards COVID-19 and how they track with their personality. The most pervasive patterns are with our measures of emotional stability and negative affect. These traits had the largest and widest correlations across attitudes towards COVID-19. These traits may be useful in the minimizing risks in one’s life including the virus. Indeed, so-called neurotic people appear to be more fearful, more trusting in others and systems likely to protect them (e.g., scientists), and less likely to trust in systems shown to not help them (e.g., prayer). Taken together, this anxious disposition may be facultatively useful at minimizing COVID-risk and maximizing their ability to get effective treatment [23]. In addition, we showed this disposition was instrumental in this way for women more than men. Another strong pattern was for those who were religious [16]. Such people were distrusting of science, thought God created the virus, and felt prayer was the best solution. While we found other associations for the Dark Triad traits and the Big Five traits, in total we highlight some of the reasons that people may be in such disagreements about what to do about the virus at the individual and institutional levels. Personality and participant’s sex all appear to play a role in the psychology of COVID-19 beliefs.

## Supporting information

S1 Table Mediation of sex differences in attitudes towards COVID-19 by personality traits as shown in direct effects and indirect effects [95% CIs] for individual traits and multiple mediation (i.e., “combined”). (DOCX)

S2 Table Mediation of national differences in attitudes towards COVID-19 by personality traits as shown in direct effects and indirect effects [95% CIs] for individual traits and multiple mediation (i.e., “combined”). (DOCX)
