# Supplementary material for: Opinions and options about COVID-19: Personality correlates and sex differences in two European countries
Source: PLoS One. 2022 Jun 3;17(6):e0268193. doi: 10.1371/journal.pone.0268193 (PMC9165842; doi:10.1371/journal.pone.0268193)
Supplement: S1 Table — (DOCX) [file pone.0268193.s001.docx]

| Table S1. Mediation of sex differences in attitudes towards COVID-19 by personality traits as shown in direct effects and indirect effects [95% CIs] for individual traits and multiple mediation (i.e., “combined”). | | | |
| --- | --- | --- | --- |
| **Dependent Variable** | **Mediators** | **Direct** | **Indirect** |
| ***Trust in others*** | Narcissism | -.56** [-.79, -.33] | -.02 [-.06, <.01] |
|  | Conscientiousness | -.61** [-.84, -.38] | .02 [<.01, .05] |
|  | Extraversion | -.58** [-.82, -.35] | <.01 [-.04, .03] |
|  | Emotional Stability | -.38* [-.62, -.15] | -.20** [-.27, -.13] |
|  | Negative Affect | -.36** [-.59, -.13] | -.22** [-.29, -.15] |
|  | *Combined* | -.23* [.47, <.01] | -.35** [-.45, -.24] |
| ***Fear of COVID*** | Narcissism | .52** [.28, .77] | .02 [<.01, .05] |
|  | Machiavellianism | .55** [.30, .79] | -.10 [-.03, .03] |
|  | Emotional Stability | .32** [.08, .57] | .22** [.31, .79] |
|  | Negative Affect | .28* [.04, .52] | .27** [.19, .35] |
|  | Religiousness | .48** [.24, .73] | .06** [.02, .10] |
|  | *Combined* | .14 [-.10, .38] | .41** [.31, .50] |
| ***Biological weapon*** | Emotional Stability | .20* [.02, .37] | .08** [.04, .12] |
|  | Conscientiousness | .27** [.10, .44] | .01 [<.01, .02] |
|  | Extraversion | .27** [.10, .45] | .09 [-.01, .03] |
|  | Negative Affect | .18* [.01, .35] | .10** [.06, .14] |
|  | Religiousness | .20* [.03, .37] | .07** [.03, .11] |
|  | *Combined* | .05 [-.11, .23] | .22** [.16, .29] |
| ***Naturally occurring*** | Emotional Stability | .33** [.16, .49] | .03* [<.01, .07] |
|  | Negative Affect | .29** [.13, .46] | .07** [.03, .10] |
|  | Religion | .33** [.16, .49] | .03** [.01, .06] |
|  | *Combined* | .27** [.10, .44] | .09** [.05, .14] |
| ***God created it*** | Narcissism | .22** [.09, .35] | .01 [<.01, .03] |
|  | Emotional Stability | .18** [.05, .31] | .05** [.02, .08] |
|  | Negative Affect | .12* [<.01, .24] | .11** [.07, .15] |
|  | Religiousness | .11 [<.01, .23] | .12** [.06, .18] |
|  | *Combined* | .02 [<.09, .14] | .20** [.14, .27] |
| ***Listen to scientists*** | Psychopathy | -.18** [-.28, -.08] | .04* [.01, .07] |
|  | Machiavellianism | -.16** [-.27, -.06] | .02* [<.01, .05] |
|  | Conscientiousness | -.14** [-.25, -.04] | .01 [<.01, .02] |
|  | Emotional Stability | -.10 [-.21, <.01] | -.03** [-.05, <.01] |
|  | Negative Affect | -.08 [-.19, .01] | -.05** [-.07, -.02] |
|  | Religion | -.10* [-.21, .<.01] | -.03** [-.04, -.01] |
|  | *Combined* | -.12* [-.22, -.20] | -.01 [-.06, .03] |
| ***Listen to the government*** | Psychopathy | .13* [.01, .25] | .03** [<.01, .05] |
|  | Machiavellianism | .12* [<.01, .24] | .03** [<.01, .06] |
|  | Extraversion | .13* [.01, .25] | .02* [<.01, .04] |
|  | Agreeableness | .14* [.02, .25] | .02* [.04, .04] |
|  | *Combined* | .09 [-.02, .21] | .07** [.04, .10] |
| ***Prayer*** | Narcissism | .39** [.21, .57] | .03* [<.01, .05] |
|  | Psychopathy | .42** [.24, .60] | <.01 [-.02, .02] |
|  | Machiavellianism | .41** [.24, .59] | .01 [-.01, .04] |
|  | Agreeableness | .38**[.20, .56] | .04* [.01, .07] |
|  | Extraversion | .43** [.25, .61] | <.01 [-.03, .02] |
|  | Negative Affect | .28** [.10, .45] | .14** [.09, .19] |
|  | Religiousness | .15** [.01, .28] | .27** [.15, .39] |
|  | *Combined* | .06 [-.07, .20] | .36** [.23, .49] |
| *Note*. *Combined* = all above traits for each DV combined. * *p* < .05, ** *p* < .01 | | | |
|  |  |  |  |
