# Supplementary material for: Opinions and options about COVID-19: Personality correlates and sex differences in two European countries
Source: PLoS One. 2022 Jun 3;17(6):e0268193. doi: 10.1371/journal.pone.0268193 (PMC9165842; doi:10.1371/journal.pone.0268193)
Supplement: S2 Table — (DOCX) [file pone.0268193.s002.docx]

| Table S2. Mediation of national differences in attitudes towards COVID-19 by personality traits as shown in direct effects and indirect effects [95% CIs] for individual traits and multiple mediation (i.e., “combined”). | | | |
| --- | --- | --- | --- |
| **Dependent Variable** | **Mediators** | **Direct** | **Indirect** |
| ***Trust in healthcare*** | Narcissism | .68** [.37, .98] | .04 [<.01, .92] |
|  | Emotional Stability | .56** [.25, .87] | .16** [.07, .24] |
|  | Negative Affect | .54** [.23, 86] | .17** [.09, .25] |
|  | Religiousness | .61** [.31, .92] | .10** [.04, .16] |
|  | *Combined* | .36* [.04, .68] | .36** [.24, 48] |
| ***Trust in others*** | Narcissism | 1.09** [.88, 1.31] | .01 [-.01, .04] |
|  | Openness | 1.14** [.92, 1.35] | -.02* [-.05, <.01] |
|  | Conscientiousness | 1.09** [.88, 1.30] | .01 [<.01, .03] |
|  | Extraversion | 1.12** [.90, 1.34] | <.01 [-.04 .02] |
|  | Emotional Stability | .91** [.68, 1.41] | .20** [.13, .27] |
|  | Negative Affect | .89** [.62, 1.10] | .22** [.16, .29] |
|  | *Combined* | .80** [.58, 1.02] | .31** [.21, .41] |
| ***Fear of COVID*** | Narcissism | -.98** [-1.21, -.07] | -0.1 [-.04, .01] |
|  | Machiavellianism | -1.02** [-1.25, -.79] | .02 [-.02, .07] |
|  | Emotional Stability | -.76** [-.98, -.53] | -.23** [-.31, -.16] |
|  | Negative Affect | -.71** [-.93, -.49] | -.28** [-.36, -.20] |
|  | Religiousness | -.93** [-1.16, -.70] | -.06** [-.10, -.02] |
|  | *Combined* | -.59** [-.82, -.36] | -.40** [-.50, -.29] |
| ***Natural occurring*** | Emotional Stability | -.17* [-.33, -.02] | -.05* [-.09, <.01] |
|  | Negative Affect | -.14 [-.29, .01] | -.08** [-.12, -.04] |
|  | *Combined* | -.14 [-.29, .01] | -.08** [-.13, -.03] |
| ***God created it*** | Narcissism | -.30** [-.42, -.18] | <.01 [-.02, <.01] |
|  | Openness | -.32** [-.44, -.20] | .01 [<.01, .02] |
|  | Conscientiousness | -.30** [-.41, -.18] | -.01 [-.02, <.01] |
|  | Emotional Stability | -.26** [-.38, -.14] | -.05** [-.09, -.01] |
|  | Negative Affect | -.19** [-.30, -.07] | -.12** [-.16, -.08] |
|  | Religiousness | -.16** [.27, -.06] | -.14** [-.19, -.09] |
|  | *Combined* | -.07 [-.18, .04] | -.31** [-.43, -.19] |
| ***Rely on luck*** | Machiavellianism | -.05 [-.18, .07] | .06** [.02, .10] |
|  | Openness | <.01 [-.13, .11] | .01* [<.01, .03] |
|  | Conscientiousness | .01 [-.10, .14] | -.01 [-.02, <.01] |
|  | Agreeableness | -.01 [-.14, .10] | .02* [<.01, .04] |
|  | Emotional Stability | .08 [-.04, .20] | -.07** [-.11, -.03] |
|  | Negative Affect | .11 [-.01, .23] | -.10** [-.14, -.06] |
|  | Religiousness | .05 [-.07, .17] | -.04** [-.06, -.02] |
|  | *Combined* | .06 [-.06, .20] | -.06 [-.12, <.01] |
| ***Listen to the government*** | Machiavellianism | -.35** [-.44, -.23] | -.03* [-.06, <.01] |
|  | Openness | -.38** [-.50, -.28] | <.01 [-.01, <.01] |
|  | Extraversion | -.37** [-.49, -.25] | -.02* [-.03, <.01] |
|  | Agreeableness | -.35** [-.47, -.23] | -.03** [-.06, -.01] |
|  | *Combined* | -.32** [-.44, -.19] | -.07** [-.10, -.03] |
| ***Listen to the media*** | Machiavellianism | -.59** [-.72, -.45] | <.01 [-.03, .01] |
|  | Openness | -.58** [-.71, -.45] | -.01 [-.02, .17] |
|  | Agreeableness | -.56** [.69, .43] | -.03** [-.06, -.01] |
|  | Religiousness | -.58** [-.71, -.45] | -.01 [-.03, <.01] |
|  | *Combined* | -.55** [-.06, -.41] | -.04* [-.08, -.01] |
| ***Prayer*** | Narcissism | .42** [-.59, -.26] | -.02* [-.05, <.01] |
|  | Machiavellianism | -.44** [-.61, -.27] | <.01 [-.04, .02] |
|  | Openness | -.47** [-.63, -.30] | <.01* [.<.01, .03] |
|  | Agreeableness | -.37** [-.54, -.20] | -.07** [-.11, -.04] |
|  | Emotional Stability | -.40** [-.57, -.23] | -.04 [-.09, .08] |
|  | Negative Affect | -.29** [-.46, -.12] | -.16** [-.21, -.10] |
|  | Religiousness | -.12* [-.24, <.01] | -.33** [-.43, -.21] |
|  | *Combined* | <.01 [-.12, .13] | -.45** [-.58, -.33] |
| *Note*. *Combined* = all above traits for each DV combined. * *p* < .05, ** *p* < .01 | | | |
|  |  |  |  |
